# Predicting gene promoter methylation in non-small-cell lung cancer by evaluating sputum and serum

**DOI:** 10.1038/sj.bjc.6603721

**Published:** 2007-04-03

**Authors:** S A Belinsky, M J Grimes, E Casas, C A Stidley, W A Franklin, T J Bocklage, D H Johnson, J H Schiller

**Affiliations:** 1 Lung Cancer Program, Lovelace Respiratory Research Institute, 2425 Ridgecrest Dr. SE, Albuquerque, NM 87108, USA; 2 Division of Internal Medicine, University of New Mexico, 2325 Camino De Salud, Albuquerque, NM 87131, USA; 3 Division of Pathology, University of Colorado, PO Box 6511, Aurora, CO 80045; 4 Division of Hematology/Oncology, Vanderbilt University, 2220 Pierce Ave, Nashville, TN 37232, USA; 5 Division of Hematology/Oncology, University of Texas Southwestern, 5323 Harry Hines Blvd, Dallas, TX 75390, USA

**Keywords:** gene promoter methylation, sputum, serum, tumour, lung cancer

## Abstract

The use of 5-methylcytosine demethylating agents in conjunction with inhibitors of histone deacetylation may offer a new therapeutic strategy for lung cancer. Monitoring the efficacy of gene demethylating treatment directly within the tumour may be difficult due to tumour location. This study determined the positive and negative predictive values of sputum and serum for detecting gene methylation in primary lung cancer. A panel of eight genes was evaluated by comparing methylation detected in the primary tumour biopsy to serum and sputum obtained from 72 patients with Stage III lung cancer. The prevalence for methylation of the eight genes in sputum (21–43%) approximated to that seen in tumours, but was 0.7–4.3-fold greater than detected in serum. Sputum was superior to serum in classifying the methylation status of genes in the tumour biopsy. The positive predictive value of the top four genes (p16, DAPK, PAX5 *β*, and GATA5) was 44–72% with a negative predictive value for these genes ⩾70%. The highest specificity was seen for the p16 gene, and this was associated with a odds ratio of six for methylation in the tumour when this gene was methylated in sputum. In contrast, for serum, the individual sensitivity for all genes was 6–27%. Evaluating the combined effect of methylation of at least one of the four most significant genes in sputum increased the positive predictive value to 86%. These studies demonstrate that sputum can be used effectively as a surrogate for tumour tissue to predict the methylation status of advanced lung cancer where biopsy is not feasible.

Lung cancer remains the leading cause of cancer-related death among men and women in the United States largely due to the lack of early diagnosis and the resistance of advanced disease to standard radiation and chemotherapy (Jemal *et al*, 2002; Schiller *et al*, 2002). These issues have prompted a renewed focus on developing targeted therapy based on pathways that are altered during the pathogenesis of lung cancer. Two targets that are being exploited are the epidermal growth receptor family and vascular endothelial growth factor (Auberger *et al*, 2006; Azim and Ganti, 2006; Dy and Adjei, 2006; Sandler and Herbst, 2006; Spicer *et al*, 2007). Although some patients have shown dramatic and sustained responses to these therapies, overall response for non-small-cell lung cancer (NSCLC) patients in Phase III trials has been modest, albeit significant increases in survival have been reported (Auberger *et al*, 2006; Azim and Ganti, 2006; Dy and Adjei, 2006; Sandler and Herbst, 2006; Spicer *et al*, 2007). Patient selection based on markers such as epidermal growth factor receptor mutation or aneuploidy is likely important for maximising the efficacy of these targeted therapies (Bunn *et al*, 2006). Unfortunately, even preselecting patients based on dysfunction within the targeted pathway is unlikely to yield sustained response in most cases due to molecular heterogeneity of lung tumours.

One theoretical approach to this problem is to reverse the life–threatening cancer phenotype to that of a less lethal cancer phenotype. This strategy is quite difficult in the setting of genes whose function has been altered through mutation or deletion, but may prove feasible for epigenetic alterations that arise during lung carcinogenesis. The silencing of genes through promoter hypermethylation is now recognised as a major and causal epigenetic event that occurs during lung cancer initiation and progression (Belinsky, 2005). Genes silenced by methylation are involved in all aspects of normal cell function that include control of cell proliferation, differentiation, and death. Gene silencing involves methylation of cytosines in the gene promoter region, removal of histone lysine tails, and other modifications of histones that culminate in the establishment of chromatin modifications that block transcription (Jones and Baylin, 2002; Herman and Baylin, 2003). Cytosine methylation appears to be dominant in transcriptional repression, and inhibitors of the cytosine DNA-methyltransferases, 5-azacytidine, and 5-deoxyazacytidine (DAC), can induce re-expression of genes silenced through promoter hypermethylation (Jones and Baylin, 2002; Herman and Baylin, 2003). Importantly, while inhibitors of histone deacetylation (HDAC) are not very effective in inducing re-expression of genes silenced by promoter hypermethylation, such inhibitors can synergise with demethylating agents to relieve transcriptional repression (Cameron *et al*, 1999). These advances have now been translated from the bench to the bedside to address whether restoring expression of genes silenced by methylation can be used therapeutically to treat cancer. Clinical trials with demethylating agents alone or in combination with HDAC inhibitors have shown promising responses in the treatment of myeloid malignancies (Gore *et al*, 2006; Yang *et al*, 2006). Specifically, treatment with 5-azacytidine followed by the HDAC inhibitor, sodium phenylbutyrate, was associated with induction of acetylation of histones H3 and H4. All responders showed cytogenetic effects and demethylation of the *p15* or *CDH-1* promoters, while nonresponders failed to show any demethylation (Gore *et al*, 2006).

The extension of this targeted approach to solid tumours such as those in the lung may also hold promise as a therapy. This supposition is supported by our recent work in which combined treatment with DAC and sodium phenylbutyrate reduced the number of developing lung tumours in a murine model by >50% (Belinsky *et al*, 2003). Similar to the strategy used in clinical trials on myeloid malignancies, it will be important to determine whether the drug combination causes demethylation of genes in the lung tumours. Direct measurement of gene-specific promoter methylation will not be feasible in many cases due to tumour location and to patient compliance for the conduct of multiple biopsies during the treatment period. An alternative strategy could be to monitor methylation in a noninvasively accessible biological fluid such as sputum or serum. The purpose of this study was to determine the positive and negative predictive values of sputum and serum for gene methylation in primary NSCLC. A panel of eight genes was evaluated by comparing methylation detected in the primary tumour biopsy to sputum and serum obtained from 72 patients with Stage III NSCLC.

## MATERIALS AND METHODS

### Subject enrollment

Subjects enrolled (*n*=72) into this study were participating in the randomised Phase III trial ‘Carboplatin, Paclitaxel, and Radiotherapy, with or without Thalidomide’ through the Eastern Coast Oncology Group (ECOG 3598). The criterion for participation in the clinical trial was newly diagnosed, histologically confirmed NSCLC that was either unresectable Stage IIIA or Stage IIIB without pleural effusion. Patients were ⩾18 years of age and had no other active malignancies. In addition, no prior chemotherapy within 5 years of enrollment onto this trial was allowed or radiation to the tumour. Following enrollment in the clinical trial, the patients were asked to participate in the correlative laboratory studies by providing sputum, blood, and allowing receipt of previously collected tumour tissue. All participants in the laboratory study signed an informed consent at the enrolling institution. The Lovelace Respiratory Research Institute (LRRI) Review Board approved the conduct of this correlative study.

Selected demographics are summarised in Table 1. Cigarette smoke exposure, pack years, and smoking duration are generally not collected as part of clinical trials and thus were unavailable.

### Sputum and blood collection and processing

On enrollment, a kit for collecting blood and sputum was sent to the participating institution. Participants were provided with a sterile specimen cup containing Saccomanno's fixative in a self-addressed return mailer (Kennedy *et al*, 1996). To increase the probability that material from deep in the lung was obtained, subjects received detailed verbal instructions by study personnel at the participating institution and written instructions on how to perform the technique. Briefly, for three consecutive mornings, patients coughed deeply, and the resulting mucous was expectorated into a cup. Two slides were prepared from the sputum sample and underwent Papanicolaou staining for morphologic examination by certified cytopathologists (Saccomanno, 1978). All sputum samples, irrespective of adequacy (see Results), were processed for methylation analysis by extensive mixing by vortex, washed once with Saccomanno solution, and stored at room temperature until analysed.

Blood (32 cc) was collected by phlebotomy from all participants into SST Vacutainer cell preparation tubes (Becton Dickinson and Company, Franklin Lakes, NJ, USA). These tubes are designed to separate serum from whole blood. Following separation, the serum fraction was centrifuged at 1500 r.p.m. to remove any contaminating mononuclear cells. The serum fraction was then frozen at −80°C until processing for DNA isolation.

### Nucleic acid isolation and methylation-specific PCR

DNA was isolated from sputum and tumour biopsies (1–15 mm, two to three sections) by protease digestion followed by phenol chloroform extraction and ethanol precipitation. Plasma DNA (10 ml) was isolated using the QIAGEN blood maxi kit (Invitrogen, Carlsbad, CA, USA). DNA was quantitated by spectrophotometer at an absorbance of 260 nm. In addition, a subset of DNA recovered from serum samples was quantitated by the DNA dip-stick test (Invitrogen). Quantitation by these two techniques differed by <5%.

Promoter methylation was assessed in the *p16*, *O*^*6*^*-methylguanine-DNA methyltransferase* (*MGMT*), *death-associated protein kinase* (*DAPK*), *ras effector homolog 1* (*RASSF1A*), *H-cadherin*, *GATA5*, *PAX5 α*, and *PAX5 β* genes. When these studies were originally initiated, methylation assays were only conducted for *p16, MGMT, DAPK*, and *RASSF1A*. The other genes were added when results from our group demonstrated their potential for predicting lung cancer risk and for early detection (Belinsky *et al*, 2005, 2006). These genes were also selected based on their prevalence in lung cancer (⩾30%) and diversity of function (Belinsky, 2004). Because of limited DNA available from some tumour biopsies and serum, we were unable to assay for methylation of the *H-cadherin*, *GATA5*, *PAX5 α*, and *PAX5 β* genes in all specimens.

Nested methylation-specific PCR (MSP) was used to detect methylated alleles in DNA recovered from tumour tissue, sputum, or serum. We used our nested MSP assay, described in detail previously (Palmisano *et al*, 2000), because of its increased sensitivity for the detection of promoter hypermethylation in biological fluids and because of the ability to perform Stage 1 multiplex PCR. The amplification of four genes in a Stage 1 PCR was needed due to the low amount of DNA recovered from the serum and tissue biopsies from some subjects. To accurately compare the prevalence for methylation in serum and sputum with a sensitivity of 1 in 10–20 000, 50–120 ng of DNA were used for Stage 1 PCR following modification with bisulphite. Because of tissue degradation from formalin fixation and storage in paraffin, sensitivity was approximately 1 in 500 even with the nested MSP approach. PCR primers for Stages 1 and 2 have been described elsewhere (Palmisano *et al*, 2000; Belinsky *et al*, 2002; Palmisano *et al*, 2003). A subset of samples (20%) that gave positive methylation products also was analysed by methylation-sensitive restriction enzyme digestion of the resulting PCR product. The restriction digestion allows one to examine the methylation state of CpGs within the amplified PCR product and serves as a control for false priming. Digestion within at least one of the restriction sites was seen for all samples, positively confirming methylation.

### Statistical methods

Categorical variables were summarised with percentages and continuous variables were summarised with medians and ranges. Each gene was examined separately, but to assess the importance of multiplicity, a methylation index was based on obtaining the number of genes that were methylated among the panel of eight genes. Because some of the samples were missing methylation data for four of the genes, a second methylation index based on the panel of four genes with complete data (*p16*, *MGMT*, *RASSF1A*, and *DAPK*) was also created. Fisher's exact test was used to compare the frequency of methylation between groups based on gender and characteristics of the sample or tumour, such as adequacy or tumour type. The two-sample *t*-test was used to compare DNA concentration in serum between methylated and unmethylated samples for each gene. Paired sputum and serum samples were compared for differences in methylation frequency with the exact form of McNemar's test. In comparisons of methylation results for sputum and serum samples with tumour samples, sensitivity, specificity, positive and negative predictive value were calculated, along with 95% confidence intervals. To further explore the association between the sputum and serum samples and the tumour sample, logistic regression was used to obtain odds ratios for tumour methylation based on methylation status of the sputum or serum. All analyses were conducted in SAS version 9.1.3.

## RESULTS

### Tumour histology, sputum cytology, and DNA recovery from serum

Adenocarcinoma comprised the major histology (40%) followed by 31% of the tumours being classified as squamous cell carcinoma (SCC, Table 1). Nineteen percent of tumours were classified as NSCLC due to insufficient amount of tissue to classify by specific histology.

Sputum adequacy defined as the presence of deep lung macrophages or Curschmann's spiral (Saccomanno, 1978) was observed for 87% of the specimens collected. Atypia ranging from mild to severe was seen in 35 of the sputum specimens (Table 1). Lung cancer diagnosed as SCC, adenocarcinoma, or NSCLC was detected in sputum from 11 of 72 cases. The median amount of DNA recovered from serum was 39 ng ml^−1^ (Table 1).

### Prevalence for gene methylation in tumour, sputum, and serum

The prevalence for methylation of the eight genes evaluated in tumours ranged from 15% for *MGMT* to 47% for the *p16* gene (Table 2). With the exception of *MGMT*, these findings approximate to that seen in previous studies for methylation of these genes in NSCLC (Belinsky, 2004). *P16* and *GATA5* were the two most common genes methylated in sputum (∼40%, Table 2). The remaining genes were methylated at prevalences from 21 to 32%. The prevalence for methylation of these genes in sputum was 0.7–4.3-fold greater than detected in serum. Significant differences (*P*<0.05) between sputum and serum were seen for methylation for all genes except *PAX5 α* and *GATA5*. There were no significant differences in prevalence for methylation in tumour or serum by gender (not shown). Interestingly, relatively more sputum samples from men were methylated for the *p16*, *PAX5 β*, and *GATA5* genes than for women (*P*< 0.05).

The influence of histology, SCC, or non-SCC on methylation in tumours and detection in sputum and serum was also assessed. Non-SCC comprised all other histological types described in Table 1. The differences seen did not reach statistical significance due to the decrease in sample size when comparing effect of histology, but were a greater prevalence for methylation of the *p16* (62 *vs* 39%, *P*=0.09), and *DAPK* genes (46 *vs* 22%, *P*< 0.05) in SCC compared to non-SCC. *P16* was also more commonly methylated in sputum from SCC than non-SCC cases (54 *vs* 33%, *P*=0.09), while the opposite scenario was observed for this gene in serum (8 *vs* 28%, *P*=0.07). The effect of adequacy and positive sputum cytology on the detection of gene methylation was also assessed. As seen in previous studies (Belinsky *et al*, 2005, 2006), no association was observed between adequacy and our ability to detect gene methylation. This likely reflects the fact that the classification of adequacy is based largely on the presence of deep lung alveolar macrophages in the sputum sample, but not the presence of epithelial cells that are shed from both the airways and alveolar regions. These cells are the source of the malignant or precancerous cells that harbour the methylated genes. Sputum specimens were divided into those that were positive for cancer or moderate/severe atypia (*n*=20) and compared to specimens where no abnormality or inadequacy was observed (*n*=25). The prevalence for individual gene methylation was always greater in the atypia/cancer group compared to the no abnormality/inadequate group (Table 3). In contrast, no association was seen between the detection of gene promoter methylation and the amount of freely circulating DNA in serum (*P*>0.1 for each gene).

### Increased multiplicity for gene methylation in sputum compared to serum

The presence of at least one, but preferably multiple biomarkers (methylated genes) in the primary tumour should increase the efficiency of monitoring the effectiveness of demethylating therapy by evaluating the biological fluid. Using the four-gene panel, at least one gene was methylated in 79% of tumours, while 15% of tumours were methylated for three or more genes (Table 2). The detection of any methylated gene in tumours improved to 89% by increasing the panel of genes examined to eight and improved detection also associated with greater than half of the tumours being positive for methylation of three or more genes. With respect to sputum, increasing the gene panel to eight reduced the number of samples that were negative for gene methylation and substantially increased the multiplicity for methylation (Table 2). Only 10 of 72 cases were negative for methylation of all genes in sputum, while three or more genes were methylated in 44% of sputum samples. In contrast, the presence of gene methylation in serum was significantly lower than in sputum. Even with the eight-gene panel, no methylated genes were detected in 44% of cases, while only 7% of cases were positive for three or more methylated genes in DNA recovered from serum (Table 2).

### Correlation of gene promoter methylation in sputum and serum with tumour methylation

Sputum was superior to serum in classifying the methylation status of genes in the tumour biopsy. Sensitivity and specificity for five genes (*p16, MGMT, DAPK, PAX5 β*, *GATA5*) ranged from 45 to 63% and 62 to 79%, respectively (Table 4). The highest specificity was seen for *p16*, and this specificity was associated with an odds ratio of six for methylation in the tumour when this gene was methylated in sputum. With the exception of *MGMT* where the methylation prevalence was lower in the tumours than sputum, the positive predictive value for the other four genes was 45–72%. The negative predictive value for these five genes was ⩾70%. In general, the sensitivity and specificity of sputum for classifying methylation of these eight genes did not differ between SCC and non-SCC. The one exception was *p16*, whereas for SCC, sensitivity and specificity was increased to 81 and 90% with a positive and negative predictive value of 93 and 75%, respectively. In contrast, for serum, the individual sensitivity for all eight genes was only 6–27%, although specificity was 73–98% (Table 4). The positive predictive value for methylation in serum exceeded 70% for two genes, *MGMT* and *RASSF1A*. Combining methylation results from sputum and serum did not significantly improve sensitivity or specificity for predicting the methylation status in the tumour biopsy.

A key question is the overall predictive power of a gene panel assayed in sputum to accurately classify the methylation status of at least one of those genes in the tumour. This was evaluated by looking at the combined effect of having methylation of *p16*, *DAPK, PAX5 β*, or *GATA5* in the sputum. These genes were selected because their individual positive and negative predictive values were superior to the other genes. The composite positive predictive value for these four genes was 86%; however, the negative predictive value was 42%. The lower negative predictive value is due largely to the fact that although 43 of the 56 tumours were methylated for at least one of these genes, the matched sputum from 11 of these methylated tumours was negative for methylation of any of the four genes.

## DISCUSSION

These studies demonstrate the superiority of sputum over serum as a surrogate for tumour tissue to predict the methylation status of advanced lung cancer where biopsy is not feasible. Gene methylation of both SCC and non-SCC tumours could be predicted through analysis of sputum substantiating the use of this fluid for monitoring both central and peripheral lung tumours. The false-positive methylation seen in sputum likely stems from the extensive field cancerisation induced by smoking and from which the lung cancer arises (Slaughter *et al*, 1953). In contrast, false negatives likely occur because of the lack of exfoliation of tumour or atypical cells into the sputum at amounts needed for detection. This conclusion is supported by the differences in prevalence seen for all eight genes evaluated for methylation between sputum samples positive for atypia or cancer *vs* samples that were cytologically normal or inadequate.

Serum proved to be a poor surrogate to predict the methylation status of the tumour, largely because of the low prevalence for detection of methylated genes in this fluid. Our gene prevalences for *p16, MGMT, DAPK*, and *RASSF1A* methylation in serum are very similar to that observed by Fujiwara *et al* (2005) in their study of serum from all stages of NSCLC and in our previous study of lung cancer survivors that examined free DNA recovered from plasma (Belinsky *et al*, 2005). Both Fujiwara *et al* (2005) and Esteller *et al* (1999) saw no association between methylation detection in serum and tumour stage. This low sensitivity is likely because the tumours are not releasing free DNA through apoptosis, or because the released DNA is too fragmented to allow detection of the methylated alleles of the interrogated genes. This conclusion is consistent with the fact that the median amount of DNA recovered from serum in this study did not differ from that recovered from lung cancer survivors, smokers, or never smokers (Belinsky *et al*, 2005). There has been a report of increased circulating DNA in blood from lung cancer patients; however, this finding was likely influenced by the blood separation protocol that did not conduct a second centrifugation step to remove contaminating mononuclear cells (Sozzi *et al*, 2001).

A key question from our study is what is the biological significance of detecting methylation of genes in the serum? The presence of methylation in serum could reflect the invasive potential of the tumour, a conclusion that will be evaluated through the clinical trial being conducted on these patients that will examine the relationship between the presence of methylation in serum and the response to therapy. For five of the eight genes, the positive predictive value in serum was ⩽40%. This probably reflects both the release of DNA from preinvasive lesions in the lungs and the contribution of other age-related diseases to the DNA pool recovered in blood. For example, in our previous study, two participating never smokers reported a past diagnosis of ovarian cancer and methylation of the *p16* gene was detected in DNA recovered from their plasma (Belinsky *et al*, 2005).

Field defects involving preneoplastic changes have been described in which histologically negative bronchial margins of resected NSCLC exhibit frequent hypermethylation changes in multiple genes that often reflect the methylation status of the tumour (Guo *et al*, 2004). In addition, our previous studies have detected gene methylation in bronchial epithelial cells obtained from cancer-free lung lobes of patients with cancer (Belinsky *et al*, 2002). This field cancerisation likely accounts for our ability to detect methylation in sputum from the majority of cases in spite of only being able to observe moderate to severe atypia (both predictive of cancer risk) or frank carcinoma in sputum from 20 of 72 cases. The presence of field cancerisation may be advantageous for monitoring the effectiveness of demethylating therapy because both tumour and lung tissue will be exposed to the therapy. In this study, *p16* proved to be the superior marker with respect to both positive and negative predictive value. This is likely due to the high prevalence (∼50% of tumours methylated) and the early stage of tumour development in which this gene is silenced by methylation (Belinsky *et al*, 1998; Belinsky, 2004). However, in order to assess response to therapy, a gene panel is needed whose composite methylation in sputum identifies the majority of tumours. A panel of four genes that included *p16, DAPK, PAX5 β*, and *GATA5* was methylated in 77% of tumours and had a combined positive predictive value of 86%. It will likely be necessary to evaluate response based not on one gene, but on a methylation index (number of genes). This is because unlike myeloid malignances where the cell population evaluated (bone marrow) is homogeneous, sputum is very heterogeneous negating the ability to detect quantitative differences in the extent of individual gene methylation by sequencing or quantitative real-time MSP. The methylation index is proving to be a good measure for predicting lung cancer risk. Our recent nested case–control study revealed that the concomitant methylation of three or more of a panel of six genes was associated with a six-fold increased risk for lung cancer (Belinsky *et al*, 2006). A sensitivity and specificity of 64% was seen for identifying incident lung cancer cases 3–18 months prior to clinical diagnosis. We are entering a new era of targeted cancer therapy in which future clinical trials will evaluate the efficacy of demethylation therapy on tumour growth and field cancerisation and the ability of the gene methylation index in sputum to predict response.

## Figures and Tables

**Table 1:** Summary of selected demographic variables

**Variable**	**Stage III lung cancer patient (*n*=72)**
Age^a^	62 (37, 80)
	
*Gender* (%)	
Male	49 (68)
Female	23 (32)
	
*Tumour histology* (%)	
Squamous	22 (31)
Adenocarcinoma	29 (40)
Adenosquamous	4 (6)
Large cell	3 (4)
Non-small-cell	14 (19)
	
*Sputum cytology* (%)	
Inadequate	9 (13)
Normal	16 (22)
Metaplasia	1 (1)
Mild atypia	26 (36)
Moderate atypia	6 (8)
Severe atypia	3 (4)
Invasive cancer^b^	11 (15)
Serum DNA (ng ml^−1^)^a^	39 (12, 148)

a Median (range).

b Invasive cancers included SCC (*n*=4), adenocarcinoma (*n*=3), and NSCLC (*n*=4).

SCC=squamous cell carcinoma; NSCLC=non-small-cell lung cancer.

**Table 2:** Prevalence of gene methylation and multiplicity in tumour, sputum, and serum

	**Tumour**	**Sputum**	**Serum**
**Gene**	**(Number positive (%))^a^**		
*p16*	34/72 (47)	29/72 (40)	15/72 (21)*
*MGMT*	11/72 (15)	23/72 (32)	4/72 (6)*
*RASSF1A*	31/72 (43)	19/72 (26)	7/72 (10)*
*DAPK*	22/72 (31)	22/72 (31)	7/72 (10)*
*HCAD*	17/56 (31)	19/72 (26)	3/53 (6)*
*PAX5 β*	22/56 (39)	15/72 (21)	3/53 (6)*
*PAX5 α*	20/56 (36)	22/72 (31)	8/45 (18)
*GATA5*	19/56 (34)	31/72 (43)	10/45 (22)
			
*Four gene panel^b^*			
0	15/72 (21)	21/72 (29)	43/72 (60)*
1	27/72 (38)	21/72 (29)	25/72 (35)
2	19/72 (26)	20/72 (28)	4/72 (6)*
⩾3	11/72 (15)	10/72 (14)	0/72 (0)
			
*Eight gene panel^c^*			
0	6/56 (11)	10/72 (14)	20/45 (44)*
1	12/56 (21)	18/72 (25)	12/45 (27)
2	9/56 (16)	12/72 (17)	10/45 (22)
⩾3	29/56 (52)	32/72 (44)	3/45 (7)*

a Sample size varied for tumour and serum due to limiting amount of DNA when additional four genes were added for methylation screening.

b *p16*, *MGMT*, *RASSF1A*, *DAPK*.

c *p16*, *MGMT*, *RASSF1A*, *DAPK*, *PAX5* *α*, *PAX5* *β*, *H-Cadherin*, *GATA5*.

* *P*< 0.05 when comparing prevalence of methylation in serum to sputum.

**Table 3:** Influence of cytology on prevalence of gene methylation in sputum

	**Atypia/cancer^a^**	**Normal/inadequate**
**Gene**	**(Number positive (%))**	
*p16*	11/20 (55)	8/25 (32)
*MGMT*	7/20 (35)	7/25 (28)
*RASSF1A*	10/20 (50)	4/25 (16)*
*DAPK*	9/20 (45)	5/25 (20)
*HCAD*	8/20 (40)	1/25 (4)*
*PAX5 β*	6/20 (30)	5/25 (20)
*PAX5 α*	9/20 (45)	3/25 (12)*
*GATA5*	11/20 (55)	7/25 (28)

a Includes the detection of moderate and severe atypia and all cancers in the sputum specimen.

* *P*< 0.01 when comparing prevalence of methylation in normal/inadequate to atypical/cancer.

**Table 4:** Evaluation of sputum and serum for predicting methylation state in tumour biopsy

			**OR**	**Sensitivity (%)**	**Specificity (%)**	**Positive predictive value (%)**	**Negative predictive value (%)**
**Gene**	**Fluid**	***N***	**(95% confidence interval)**				
*p16*	Sputum	72	6.1 (2.1, 17.2)	62 (45, 78)	79 (66, 92)	72 (56, 89)	70 (56, 83)
	Serum	72	0.7 (0.2, 2.2)	18 (5, 30)	76 (63, 90)	40 (15, 65)	51 (38, 64)
							
*MGMT*	Sputum	72	2.0 (0.5, 7.4)	45 (16, 75)	70 (59, 82)	22 (5, 39)	88 (79, 97)
	Serum	72	22.5 (2.1, 243.2)	27 (1, 54)	98 (95, 100)	75 (33, 100)	88 (81, 96)
							
*RASSF1A*	Sputum	72	1.3 (0.4, 3.6)	29 (13, 45)	76 (62, 89)	47 (25, 70)	58 (45, 72)
	Serum	72	3.7 (0.7, 20.8)	16 (3, 29)	95 (89, 100)	71 (38, 100)	60 (48, 72)
							
*DAPK*	Sputum	72	2.6 (0.9, 7.6)	45 (25, 66)	76 (64, 88)	45 (25, 66)	76 (64, 88)
	Serum	72	0.9 (0.2, 5.0)	9 (0, 21)	90 (82, 98)	29 (0, 62)	69 (58, 80)
							
*HCAD*	Sputum	56	1.2 (0.3, 4.3)	29 (8, 51)	74 (61, 88)	33 (9, 57)	71 (57, 85)
	Serum	48	2.5 (0.1, 43.7)	7 (0, 21)	97 (91, 100)	50 (0, 100)	72 (59, 85)
							
*PAX5 α*	Sputum	56	1.0 (0.3, 3.4)	23 (5, 40)	76 (62, 91)	38 (12, 65)	60 (46, 75)
	Serum	48	1.0 (0.1, 11.9)	6 (0, 18)	94 (85, 100)	33 (0, 87)	67 (53, 80)
							
*PAX5 β*	Sputum	56	2.5 (0.8, 7.8)	45 (23, 67)	75 (61, 89)	50 (27, 73)	71 (57, 85)
	Serum	45	0.6 (0.1, 3.5)	13 (0, 31)	80 (66, 94)	25 (0, 55)	65 (49, 80)
							
*GATA5*	Sputum	56	2.8 (0.9, 8.8)	63 (41, 85)	62 (47, 78)	46 (27, 65)	77 (62, 92)
	Serum	45	0.4 (0.1, 2.3)	13 (0, 31)	73 (58, 89)	20 (0, 45)	63 (47, 79)

OR=odds ratio.
